# Supplementary material for: Next-visit prediction and prevention of hypertension using large-scale routine health checkup data
Source: PLoS One. 2024 Nov 13;19(11):e0313658. doi: 10.1371/journal.pone.0313658 (PMC11560048; doi:10.1371/journal.pone.0313658)
Supplement: S1 Appendix — For convenience, we categorize the features into several groups, with the dimensionality denoted after each group name. The actual range of values for each feature depends on the type of physical examination or the design of the questionnaire. (PDF) [file pone.0313658.s001.pdf]

The 266 features for one visit (listed according to the practical implementation order of dimensions). For convenience, we categorize the features into several groups, with the dimensionality denoted after each group name. The actual range of values for each feature depends on the type of physical examination or the design of the questionnaire.

- Gender (2): is male, is female.
- Are your relatives have hypertension (1).
- Blood type (6): unfilled, is type A, is type B, is type O, is type AB, unknown.
- Smoking habit (4): do you smoke, how many years do you smoke, how many years have you quit smoking, how many cigarettes do you smoke per day.
- Alcohol drinking habit (8): do you drink, how many cups do you drink each time, how many years do you drink, do you drink alcohol with less then 15% content, do you drink alcohol with 15% to 30% content, do you drink alcohol with 30% to 45% content, do you drink alcohol with more then 45% content, alcohol gram-equivalent.
- Betel nuts chewing habit (4): do you chew betel nuts, how many years do you chew betel nuts, how many betel nuts do you chew per day, how many years have you quit chewing betel nuts.
- Types of nutritional supplements or health foods taken (14): no, vitamin C, vitamin E, calcium, ferrum, multivitamins, chitin, plant fibres, lecithin, propolis, algae, pollen, acidophilus, Chinese herbal medicine.
- Sports habit (5): intensity, frequency, recent duration, do you feel shortness of breath, exercise equivalent.
- Are there sources of pollution in the workplace (3): solvent, asbestos, radiation.
- Living habit (4): vegetarian diet, intensity of work, sleep hours, current health perceiving.
- ***Medical and medication history (87): has weight decreased by more than 4 kilograms in the last three months, has drug allergy, has tinnitus, has dizziness, has difficulty swallowing, is there blood in the sputum, have been coughing non-stop for the past month, is there a lump in neck, had sores or white spots in mouth continuously for a month, have a sore or lump that has not healed for over a

month, experience chest pain when not exercising, experience chest pain when exercising, have an unusual loss of appetite, does stomach hurt when hungry, does stomach hurt after eating, has blood in stool recently, is stool black recently, have bowel habits changed suddenly recently, have blood in urine recently, has the frequency of urination increased, already gone through menopause (use "no" for male), age at menopause (use 0 for male), have bone or joint issues, had any brain surgery, had any eye surgery, had any ear (or nose or throat) surgery, had asthma, long-term use of asthma medication, had chronic obstructive pulmonary disease, had any lung surgery, had anemia, is there a family history of anemia, had stroke, is there a family history of stroke, had cardiovascular disease, is there a family history of cardiovascular disease, long-term use of heart disease medication, had any heart surgery, long-term use of hyperlipidaemia medication, had diabetes, long-term use of diabetes medication, is there a family history of diabetes, had hepatitis, had liver cirrohsis, had gallbladder or bile duct surgery, had peptic ulcer disease, had hemorrhoid, long-term use of gastrointestinal medication, had any stomach surgery, had any surgery of digestive organs other than the stomach, has an appendectomy been performed, had kidney disease, had any kidney surgery, had urinary system stones, had prostate surgery (use "no" for female), had any gynecology surgery (use "no" for male), had arthritis, had gout, had any bone surgery, had thyroid disease, long-term use of thyroid medication, had any chest surgery, had any thyroid surgery, had nasopharyngeal carcinoma, is there a family history of nasopharyngeal carcinom, had liver cancer, is there a family history of liver cancer, had gastric cancer, is there a family history of gastric cancer, had lung cancer, is there a family history of lung cancer, had rectal cancer, is there a family history of rectal cancer, had cervical cancer (use "no" for male), had prostate cancer (use "no" for female), had breast cancer, is there a family history of breast cancer, had cancer (other than above), is there a family history of cancer (other than above), long-term use of traditional Chinese medication, long-term use of sedatives or sleeping pills, long-term use of uric acid medication, long-term use of steroid, long-term use of psychiatric medication, long-term use of hormones medication, long-term use of painkiller, long-term use of over-the-counter medication.

- Suspected or diagnosed cardiovascular disease (20): suspected left ventricular enlargement, left ventricular enlargement, suspected marginal left ventricular hypertrophy, marginal left ventricular hypertrophy, suspected mild left ventricular hypertrophy, mild left ventricular hypertrophy, suspected moderate left ventricular hypertrophy, suspected moderate left ventricular hypertrophy, suspected severe left ventricular hypertrophy, severe left ventricular hypertrophy, suspected left ventricular thickening, left ventricular thickening, curvature of the thoracic aorta, curvature of the thoracic aorta

with calcification, cardiomegaly with curvature of the thoracic aorta, calcification of the aortic arch, suspected curvature of the thoracic aorta, suspected curvature of the thoracic aorta with calcification, suspected cardiomegaly with curvature of the thoracic aorta, suspected calcification of the aortic arch.

- Basic body measurements (13): age, body height, body weight, body mass index, body fat, waist circumference, hip circumference, waist-hip ratio, pulse, respiration rate, chest circumference, inspiratory chest circumference, is lower limb edema.
- Routine blood tests (9): leukocyte, erythrocyte, hemoglobin, hematocrit, mean corpuscular volume, mean corpuscular hemoglobin, mean corpuscular hemoglobin, red blood cell volume distribution width, platelet.
- White blood cell classification (5): neutrocyte, lymphocyte, monocyte, eosinophil, basophil.
- Fasting blood glucose (1).
- Liver and gallbladder function tests (12): total bilirubin, direct bilirubin, total protein, albumin, albumin / globulin, globulin, alkaline phosphatase, serum glutamate oxaloacetate transaminase (SGOT), serum glutamate pyruvate transaminase (SGPT), r-glutamyltransferase, lactate dehydrogenase, SGOT / SGPT.
- Kidney function tests (3): blood urea nitrogen, creatinine, estimated glomerular filtration rate.
- Uric acid (1).
- Blood lipid tests (6): triglycerides, total cholesterol (CHOL), high density lipoprotein cholesterol (HDLC), low density lipoprotein cholesterol, CHOL/HDLC, CHOL-HDLC.
- Calcium (1).
- Phosphorus (1).
- Serum iron (1).
- Hepatitis B tests (2): surface antigen value, surface antibody value.
- Tumor marker tests (2): alpha-fetoproteinm, carcinoembryonic antigen.
- Thyroid function screening (2): free thyroxine, thyroid-stimulating hormone.
- Tissue inflammation screening (2): C-reactive protein, rheumatoid factor.

- Routine urine test (11): leukocytes, appearance, urine protein, urine glucose, bilirubin, urobilinogen, urine occult blood, ketone, nitrite, urine specific gravity, pH.
- Abdominal ultrasound (8): is liver normal, is intrahepatic cholangiocarcinoma normal, is common bile duct normal, is gallbladder normal, is kidney normal, is hepatic portal vein normal, is pancreas normal, is spleen normal.
- X-ray (2): is chest X-ray normal, is abdominal X-ray normal.
- Electrocardiogram (1): normal or abnormal.
- Otolaryngology examination (6): is ear normal, is nose normal, is throat normal, is nasopharynx normal, is oropharynx normal, is neck normal.
- Pulmonary function test (3): forced vital capacity, forced expiratory volume in 1 sec, maximum mid-expiratory flow.
- Hearing test (2): left ear, right ear.
- Ophthalmology examination (7): unaided visual acuity of the right eye, unaided visual acuity of the left eye, is color blindness, is squint, is astigmatism, left eye intraocular pressure, right eye intraocular pressure.
- Bone density screening (1).
- Blood pressure-related values (6): has hypertension, is pulse pressure difference ≥ 60, mean arterial pressure ($SBP \times 1/3 + DBP \times 2/3$), pulse pressure difference, SBP, DBP.
