# Supplementary material for: Next-visit prediction and prevention of hypertension using large-scale routine health checkup data
Source: PLoS One. 2024 Nov 13;19(11):e0313658. doi: 10.1371/journal.pone.0313658 (PMC11560048; doi:10.1371/journal.pone.0313658)
Supplement: Attachment — Submitted filename: PLOS ONE Response.pdf [file pone.0313658.s004.pdf]

Response to Journal Requirements and Reviewers' Comments

Journal Requirements: Please review your reference list to ensure that it is complete and correct. If you have cited papers that have been retracted, please include the rationale for doing so in the manuscript text, or remove these references and replace them with relevant current references. Any changes to the reference list should be mentioned in the rebuttal letter that accompanies your revised manuscript. If you need to cite a retracted article, indicate the article's retracted status in the References list and also include a citation and full reference for the retraction notice.
Response: After carefully reviewing our reference list, we confirm that none of the cited papers or materials have been retracted.

Comments (Reviewer #1): I have one remaining concern regarding Figure 8. In the figure, both XGBoost and LightGBM are plotted on a scale that starts from 0 and ends at 1000, while the scale for Random Forest begins at 600 and ends at 1000. This discrepancy makes it difficult to compare Random Forest with the other two models. I recommend adjusting the scales to be consistent across all three models to facilitate a more accurate comparison. Subject to this change, I recommend the acceptance of the manuscript.
Response: We have adjusted the scale of the X-axis of the third subplot to [0, 1000]. Additionally, to ensure that the experimental results for Random Forest (RF) are not missing in the [0, 500] region, we conducted the corresponding experiments, and updated the content in the "Results: Cross Validation" and the "Experimental Setup" subsections of the "Experimental Results and Discussion" section, along with table 3. We have also found that we mistakenly named the files for Figures 7 and 8, and we have corrected the file names in the revised version.

Comments (Reviewer #2): The authors have catered for all of my comments. They didn't respond to all, but the changes are there.
Response: We have provided additional details in the "Results: Comparing with Baseline Method," "Results: Model Performance Using Different Factors," and "Results: Use of Models for Hypertension Prevention" subsections within the "Experimental Results and Discussion" section.
